# Nicotine Supplementation Does Not Influence Performance of a 1h Cycling Time-Trial in Trained Males

**DOI:** 10.3389/fphys.2019.00292

**Published:** 2019-03-26

**Authors:** Toby Mündel, Stuart D. Houltham, Matthew J. Barnes, Stephen R. Stannard

**Affiliations:** School of Sport, Exercise and Nutrition, Massey University, Palmerston North, New Zealand

**Keywords:** smokeless tobacco, stimulant, performance, competitive, athlete, doping, WADA

## Abstract

The use of nicotine amongst professional and elite athletes is high, with anecdotal evidence indicating increased prevalence amongst cycling sports. However, previous investigations into its effects on performance have not used high-validity or -reliability protocols nor trained cyclists. Therefore, the present study determined whether nicotine administration proved ergogenic during a ∼1 h self-paced cycling time-trial (TT). Ten well-trained male cyclists (34 ± 9 years; 71 ± 8 kg; 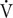
O_2_max: 71 ± 6 ml ⋅ kg^−1^ ⋅ min^−1^) completed three work-dependent TT following ∼30 min administration of 2 mg nicotine gum (GUM), ∼10 h administration of 7 mg ⋅ 24 h^−1^ nicotine patch (PAT) or color- and flavor-matched placebos (PLA) in a randomized, crossover, and double blind design. Measures of nicotine’s primary metabolite (cotinine), core body temperature, heart rate, blood biochemistry (pH, HCO_3_^−^, La^−^) and Borg’s rating of perceived exertion (RPE) accompanied performance measures of time and power output. Plasma concentrations of cotinine were highest for PAT, followed by GUM, then PLA, respectively (*p* < 0.01). GUM and PAT resulted in no significant improvement in performance time compared to PLA (62.9 ± 4.1 min, 62.6 ± 4.5 min, and 63.3 ± 4.1 min, respectively; *p* = 0.73), with mean power outputs of 264 ± 31, 265 ± 32, and 263 ± 33 W, respectively (*p* = 0.74). Core body temperature was similar between trials (*p* = 0.33) whilst HR averaged 170 ± 10, 170 ± 11, and 171 ± 11 beats ⋅ min^−1^ (*p* = 0.60) for GUM, PAT, and PLA, respectively. There were no differences between trials for any blood biochemistry (all *p* > 0.46) or RPE with mean values of 16.7 ± 0.9, 16.8 ± 0.7, and 16.8 ± 0.8 (*p* = 0.89) for GUM, PAT, and PLA, respectively. In conclusion: (i) nicotine administration, whether via gum or transdermal patch, did not exert an ergogenic or ergolytic effect on self-paced cycling performance of ∼1 h; (ii) systemic delivery of nicotine was greatest when using a transdermal patch; and (iii) nicotine administration did not alter any of the psycho-physiological measures observed.

## Introduction

Presently, the use of nicotine or nicotine-containing substances is not banned by the World Anti-Doping Agency (WADA). Yet, use of nicotine or nicotine-containing substances amongst elite and professional athletes is high and increasing. For example, cross-sectional, self-report data indicate a 25–35% prevalence of smokeless tobacco use, whilst data from anti-doping urine analyses display a detection of nicotine or its metabolites in 23–36% of samples (see Mündel, 2017 for review). Following this, WADA placed nicotine on its Monitoring Program (World Anti-Doping Agency [WADA], 2012) to further detect patterns of use to determine whether it should be upgraded to the List of Prohibited Substances.

Anecdotal reports indicate an increased prevalence of nicotine use in cycling sports. Through its psychostimulatory and sympathomimetic properties, nicotine exerts psychological and physiological effects that should be nootropic and ergogenic (Mündel, 2017). To date, eight studies have assessed performance using cycling protocols in response to consumption of nicotine or smokeless tobacco. Three studies identified an ergogenic effect (Mündel and Jones, 2006; Johnston et al., 2018; Zandonai et al., 2018) whilst the remaining five found no effect, ergogenic or ergolytic (Baldini et al., 1992; Pysny et al., 2015; Fogt et al., 2016; Zandonai et al., 2016; Mündel et al., 2017). However, the protocols used have minimal validity (time-to-exhaustion, 30 s Wingate, and incremental maximal tests) and together with the untrained, non-cyclist cohorts used reduce the reliability of these performance tests, thereby limiting the smallest worthwhile effect that can be detected (i.e., sensitivity; see Currell and Jeukendrup, 2008 for review).

The 40 km time-trial (TT) in cycling is often viewed as the blue riband event, featuring in national, international (e.g., Grand Tours), world and Olympic championships. Coyle et al. (1991) demonstrated that in well-trained and familiarized male cyclists, 40 km TT performance is highly correlated to a self-paced, 1-h laboratory cycle ergometer test. Furthermore, this simulated cycling TT has demonstrated high reliability, especially when trained, and familiarized cyclists are used as participants (Jeukendrup et al., 1996). Therefore, the primary purpose of the present study was to determine whether nicotine proved ergogenic when using a protocol and participants that have demonstrated high validity and reliability, in order to be able to translate these results to competitive cyclists and other endurance athletes.

Nicotine is delivered via different routes using a variety of products, which apart from affecting ease-of-use, can result in different nicotine bioavailability and pharmacokinetics (Mündel, 2017). Therefore, over-the-counter products such as nicotine gum, transdermal patches, inhalers and sublingual tablets will vary in their delivery of nicotine and their subsequent systemic effect due to differences in absorption etc. (Mündel, 2017). For example, use of nicotine gum results in an earlier but lower peak blood concentration of nicotine than a transdermal patch, with the former more appropriate for an acute delivery of nicotine (Mündel, 2017). To our knowledge, no previous investigation has determined any differential effect between nicotine delivery systems on exercise performance. Therefore, this was the secondary purpose of the present study.

## Materials and Methods

### Ethics Statement

The study was approved by the Central Regional Health and Disability Ethics Committee (CEN/08/09/056), and conformed to the standards set by the latest revision of the *Declaration of Helsinki*, except for registration in a database, with each participant providing informed, written consent.

### Participants

Ten well-trained male cyclists (mean ± standard deviation age: 34 ± 9 years, body mass: 71 ± 8 kg) volunteered to participate in this study. All participants were competing at a club or national level on a regular basis and maintained a weekly training volume of more than 200 km. According to De Pauw et al. (2013), our participants were classified as performance levels 3/4, or a trained/well-trained participant group due to their peak aerobic power (346 ± 46 W and 4.9 ± 0.5 W ⋅ kg^−1^) and peak rate of O_2_ consumption (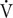
O_2_peak, 5.0 ± 0.6 L ⋅ min^−1^, and 71 ± 6 mL ⋅ kg^−1^ ⋅ min^−1^). All participants were non-smokers, and did not habitually use any form of nicotine administration.

### Experimental Overview

All participants attended the laboratory on five occasions: (1) preliminary submaximal and maximal tests, (2) experimental familiarization, and (3–5) experimental trials. The three experimental trials were completed in a randomized, crossover, double blind design. All visits were separated by 7 days, conducted at the same time of day (±1 h), following >24 h of dietary and exercise control, with participants also having refrained from alcohol and caffeine during this period. All exercise was on an electromagnetically braked cycle ergometer (Lode Excalibur, Netherlands) with participant-specific set up for the seat, handle bars and pedals, which was maintained constant for each trial within a participant. All testing was conducted in a temperate laboratory environment (18–22°C) with a fan-generated airflow of 19 km ⋅ h^−1^ facing participants.

### Preliminary Testing and Familiarization

Following body mass (Jandever, Taiwan) and height (Seca, Germany) measurements, participants began a submaximal test that consisted of four consecutive 5-min power outputs: 100, 150, 200, and 250 W, at a self-selected but constant cadence. Following 5 min active recovery and 5 min inactive recovery, a ramp protocol was used to determine 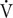
O_2_peak. Work rate began at 100 W and consisted of a linear increase at 40 W ⋅ min^−1^ until volitional fatigue. Expired gases were collected continuously (VacuMed Vista Turbofit, United States) for the determination of ventilation and O_2_ uptake (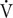
O_2_). Following this, a linear relationship between the mean rate of 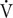
O_2_ during the last 2 min of each submaximal stage and power output was determined and used to calculate a power output which would elicit 80% of 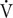
O_2_peak for each participant for the remaining TTs.

The familiarization trial was undertaken to ensure participants were accustomed to the experimental procedures and to minimize learning effects. This trial replicated entirely the experimental trial outlined below.

### Dietary and Exercise Control

Participants were asked to refrain from exercise between 24 and 48 h prior to each experimental trial. Twenty-four hours prior to each experimental trial, participants attended the laboratory to complete a standardized training ride 60 min in duration at a fixed power output that elicited ∼60% 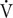
O_2_peak. Participants were then provided with a standardized snack (1× Sanitarium UP&GO, New Zealand: 823 kJ providing 30.3 g carbohydrate, 8.3 g protein, and 3.8 g fat), and recorded their diet during the 24 h period prior to the first experimental trial. This diet was replicated for each subsequent experimental trial, and in order to further minimize variation in pre-trial metabolic state a standardized meal (1× Sanitarium UP&GO, New Zealand: 823 kJ providing 30.3 g carbohydrate, 8.3 g protein and 3.8 g fat, and 1× One Square Meal, New Zealand: 1450 kJ providing 45.1 g carbohydrate, 8.4 g protein, and 11.7 g fat) was consumed 3 h prior to arriving at the laboratory for the experimental trial, after which no food was consumed. Fluid was encouraged and *ad libitum* until 3 h prior to the experimental trial.

### Nicotine/Placebo and Temperature Pill Administration

Approximately 10 h prior to each experimental trial, a staff member not involved with the research project placed a patch on the participant between the right shoulder blade and the spine. The patch was either a nicotine patch (7 mg 24 h^−1^, Habitrol, Novartis, New Zealand) or a placebo patch (orthoptic eye patch 63.5 mm × 45.7 mm, Nexcare, 3M, New Zealand). Participants were also given a factory-calibrated temperature-sensing radio pill (CorTemp^TM^, HQ Inc., United States) to ingest at this time. For most, this occurred ∼1 h before each participant went to bed. Approximately 10 h later, at 40 min prior to the beginning of the trial, the same independent staff member handed participants a piece of gum to chew for 30 min. The gum was either nicotine gum (Nicorette 2 mg, Johnson & Johnson, New Zealand) or a placebo gum (Juicy Fruit, Wrigley Corp, IL, United States). Participants were asked to chew the gum as directed by the manufacturer; briefly, this involved participants chewing the gum until the flavor became strong (∼1 min), then placing against the cheek until the flavor disappeared (∼2 min). This process was repeated until 30 min had elapsed. Participants were not aware of the research hypotheses, and were informed that the purpose of the study was to investigate the timing of nicotine administration, hence they would be administered three of the following four options: (i) PAT-GUM, PAT-PLA, PLA-GUM, PLA-PLA. Following the third experimental trial, participants were fully de-briefed. The independent staff member was only aware that they were administering intervention A (*PAT*), B (*PLA patch*), C (*GUM*), or D (*PLA gum*) with results remaining blinded to the authors until data collection was complete, after which disclosure was made.

### Experimental Procedure

Following the pre-trial control described above, participants arrived at the laboratory and were checked that they still had the radio pill in their gastro-intestinal tract. A blood sample was obtained from the antecubital vein (see below), following which participants changed into their cycling shorts and top, shoes and socks. They then received their chewing gum and rested seated for 30 min before another blood sample was obtained. Participants then completed 3 min cycling at each of 100, 150, and 200 W, to allow sufficient warm-up. Immediately on completion of the 200 W bout, the ergometer was set to linear mode based on the formula of Jeukendrup et al. (1996), where participants were required to complete an individualized set amount of work (996 ± 132 kJ) as quickly as possible, which was calculated as the equivalent of 60 min of cycling at 80% 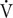
O_2_peak. Participants were notified of their progress at each 20% of the total work completed, with no other feedback provided. A 7% glucose polymer drink was provided to the participants at a rate of 100 ml every 20% of work completed and was required to be ingested within the time taken to complete 20% of work; this drink minimized the likelihood of dehydration or hypoglycemia influencing the results, and mimics competition. Immediately following the self-paced TT, participants began a 5-min cool-down (100 W) before a final blood sample was obtained.

Measurements taken during the final 2 min of each 20% work completed included heart rate (Polar Vantage XL, Polar Electro), gastro-intestinal body temperature (T_gi_), Borg’s rating of perceived exertion (RPE) measured using the 15-grade scale, from 6 to 20 (Borg, 1970), and work completed.

### Blood Sampling and Analyses

Venous blood samples were obtained from an antecubital vein into two vacutainer tubes (Becton-Dickinson, Plymouth, United Kingdom), one 4 ml containing lithium heparin and one 4 ml containing clot activator. Following inversion, the tube containing clot activator was allowed to clot at room temperature for 30 min before being centrifuged (Eppendorf, Hamburg, Germany) at 4°C for 10 min at 805 *g*. Serum was removed, aspirated into 500 μl aliquots and frozen at −80°C for later analyses using high-performance liquid chromatography (HPLC). The tube containing lithium heparin was analyzed within 30 min for determination of pH, bicarbonate and lactate via an automated analyzer (Radiometer, Brønshøj, Denmark).

Due to nicotine’s tendency to fluctuate and relatively short half-life, cotinine, its major metabolite with a longer retention time is preferred, especially for anti-doping purposes (Dhar, 2004; Mündel, 2017). Sample preparation, extraction and analysis by HPLC were based on previous methodology (Massadeh et al., 2009) and performed in duplicate. The HPLC system (Shimadzu Prominence 20 Series) consisted of a DGU-20AS Prominence degasser, SIL-20AC Autosampler, SPD-M20A Diode array detector and a CTA-20A column oven with a Phenomenex Luna 5 μ C18 (2) 100A 150 mm × 4.6 mm column attached. Operating conditions were as per the method used by Massadeh et al. (2009) except for the column, with a limit of detection for cotinine of 7.8 ng ⋅ mL^−1^.

### Data and Statistical Analyses

All descriptive and statistical analyses were performed with SPSS software for windows (IBM SPSS Statistics 20, NY, United States). Descriptive values were obtained and reported as means and standard deviation (SD) unless stated otherwise. Levene’s test was used to ensure data did not differ substantially from a normal distribution. Data were analyzed using two-way (treatment × time) ANOVA for repeated measures. Sphericity was assessed and where the assumption of sphericity could not be assumed, adjustments to the degrees of freedom were made (ε > 0.75, Huynh-Feldt; ε < 0.75, Greenhouse-Geisser). Where main or interaction effects occurred, *post hoc* pairwise analyses were performed using a paired samples *t*-test (Bonferroni correction where relevant), with statistical significance set at *P* ≤ 0.05. Partial eta-squared (η_p_^2^) is reported as a measure of effect size, with demarcations of small (<0.09), medium (>0.09 and <0.25), and large (>0.25) effects, respectively (Cohen, 1988). This combination of statistical significance and effect size provided an indication of the likelihood of committing a Type I (i.e., *P* ≤ 0.05 but η_p_^2^ < 0.09) or II (i.e., *P* < 0.10 but η_p_^2^ > 0.25) error. The typical error of measurement as a coefficient of variation (CV) between trials was calculated according to Hopkins (2000). Finally, we sought to determine whether [cotinine] was associated with exercise performance and body mass, using Pearson’s correlation coefficient to describe the form and strength of bivariate association for absolute values.

## Results

### Treatment Verification

**FIGURE 1:**
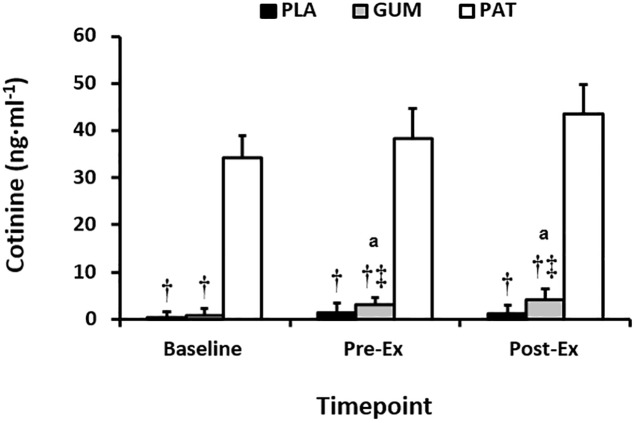
Serum cotinine at baseline, before (Pre-Ex) and following (Post-Ex) the time-trial for placebo (PLA), nicotine gum (GUM), and nicotine patch (PAT) treatments. Values are mean (SD) for *n* = 10. † indicates significantly different to PAT, ‡ indicates significantly different to PLA, and ^a^ indicates significantly different to corresponding baseline value.

Plasma cotinine concentrations can be seen in Figure 1. Main effects of treatment (*p* < 0.01, η_p_^2^ = 0.85) and time (*p* = 0.03, η_p_^2^ = 0.31) but no interaction (*p* = 0.27, η_p_^2^ = 0.14) were observed, such that the magnitude in concentrations were attained in the following order: PAT > GUM > PLA, and concentrations increased above baseline for GUM whilst concentrations remained constant for PAT and PLA. Participants reported no adverse effects with overnight exposure to nicotine via the transdermal patch or through chewing gum.

### Time-Trial Performance

**FIGURE 2:**
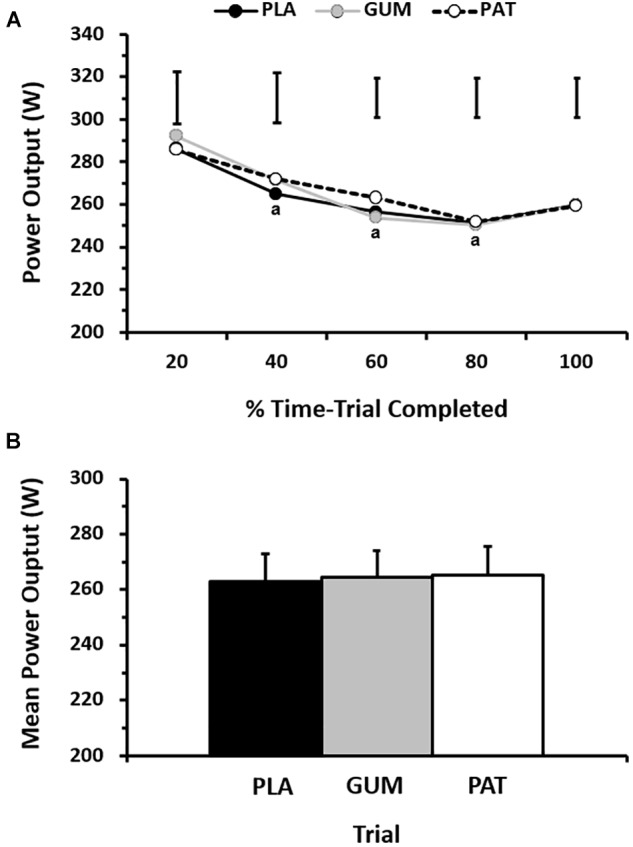
Power output for every 20% completed during the time-trial **(A)**, and mean power output across the time-trial **(B)** for placebo (PLA), nicotine gum (GUM), and nicotine patch (PAT) treatments. Values are mean (SD) for *n* = 10. ^a^ indicates significantly different to corresponding baseline value.

Mean power output between treatments and the profile over time can be seen in Figure 2. The self-paced power output profile was similar between treatments (*p* = 0.71, η_p_^2^ = 0.04) but changed over time (*p* < 0.01, η_p_^2^ = 0.65) such that power output decreased with time before a characteristic end-spurt; however, this was not dependent on treatment (interaction: *p* = 0.27, η_p_^2^ = 0.15). Time to complete the set work was similar between treatments (*p* = 0.73, η_p_^2^ = 0.03) with performance times of 63.3 ± 4.1 min, 62.9 ± 4.1 min, and 62.6 ± 4.5 min for PLA, GUM and PAT, respectively. This corresponded to mean power outputs of 263 ± 33, 264 ± 31, and 265 ± 32 W, respectively (*p* = 0.74, η_p_^2^ = 0.03).

When viewing the performance trials by completion order, the typical error of measurement as a CV between trials was 2.5 ± 1.2%. By comparison, the change in performance time due to treatments was −0.6 ± 4.4% (GUM) and −1.0 ± 4.8% (PAT).

### Physiological and Perceptual Responses

**FIGURE 3:**
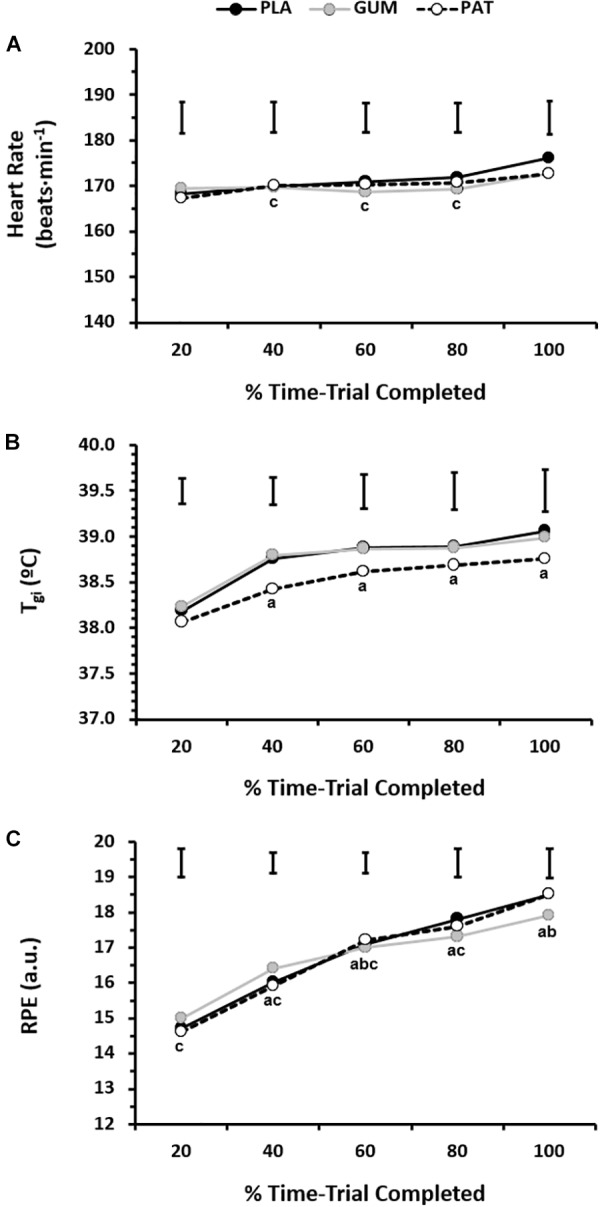
Heart rate (**A**, *n* = 10), gastro-intestinal temperature (**B**, *n* = 9), and rating of perceived exertion (**C**, *n* = 10) for every 20% completed during the time-trial for placebo (PLA), nicotine gum (GUM), and nicotine patch (PAT) treatments. Values are mean (SD). ^a^ indicates significantly different to corresponding 20% value, ^b^ indicates significantly different to corresponding previous value, and ^c^ indicates significantly different to corresponding 100% value.

The responses for heart rate, core temperature and perceived exertion can be seen in Figure 3. The heart rate response was similar between treatments (*p* = 0.60, η_p_^2^ = 0.06) but changed over time (*p* = 0.01, η_p_^2^ = 0.51) such that heart rate was maintained (∼170 beats ⋅ min^−1^) until it increased (∼174 beats ⋅ min^−1^) with the end-spurt; however, this was not dependent on treatment (interaction: *p* = 0.19, η_p_^2^ = 0.16). The core temperature response was similar between treatments (*p* = 0.32, η_p_^2^ = 0.15) but changed over time (*p* = 0.01, η_p_^2^ = 0.57) such that core temperature increased until 40% before reaching a relative plateau, with end-exercise values of 38.9 ± 0.7°C; however, this was not dependent on treatment (interaction: *p* = 0.72, η_p_^2^ = 0.05). The RPE was similar between treatments (*p* = 0.89, η_p_^2^ = 0.01) but changed over time (*p* < 0.01, η_p_^2^ = 0.77) such that RPE increased progressively from 14.7 ± 1.1 (a.u.) to 18.3 ± 1.2 (a.u.); however, this was not dependent on treatment (interaction: *p* = 0.15, η_p_^2^ = 0.17).

### Blood Biochemical Responses

The responses for pH, bicarbonate and lactate can be seen in Table 1. The pH was similar between treatments (*p* = 0.99, η_p_^2^ < 0.01) but changed over time (*p* < 0.01, η_p_^2^ = 0.73) such that pH was decreased following exercise; however, this was not dependent on treatment (interaction: *p* = 0.80, η_p_^2^ = 0.05). The bicarbonate response was similar between treatments (*p* = 0.46, η_p_^2^ = 0.09) but changed over time (*p* < 0.01, η_p_^2^ = 0.95) such that bicarbonate was decreased following exercise; however, this was not dependent on treatment (interaction: *p* = 0.78, η_p_^2^ = 0.05). The lactate response was similar between treatments (*p* = 0.89, η_p_^2^ = 0.01) but changed over time (*p* < 0.01, η_p_^2^ = 0.90) such that lactate decreased from baseline to pre-exercise and then increased following exercise; however, this was not dependent on treatment (interaction: *p* = 0.36, η_p_^2^ = 0.12).

**Table 1:** Measures of venous pH, bicarbonate (HCO_3_**^−^**), lactate (La^−^) for placebo (PLA), nicotine gum (GUM), and nicotine patch (PAT) treatments.

	PLA			GUM			PAT		
	Baseline	Pre-Ex	Post-Ex	Baseline	Pre-Ex	Post-Ex	Baseline	Pre-Ex	Post-Ex
pH (a.u.)	7.57 (0.07)	7.59 (0.08)	7.49 (0.06)^ab^	7.58 (0.08)	7.59 (0.05)	7.50 (0.09)^ab^	7.56 (0.08)	7.58 (0.07)	7.51 (0.06)^ab^
HCO_3_^−^ (mmol ⋅ l**^−^**^1^)	29.8 (2.3)	29.1 (1.1)	21.1 (2.4)^ab^	29.6 (2.2)	30.0 (1.9)	20.9 (3.3)^ab^	30.6 (2.5)	30.5 (2.6)	22.0 (2.9)^ab^
La^−^ (mmol ⋅ l**^−^**^1^)	1.3 (0.9)	1.1 (0.2)^a^	5.0 (1.6)^ab^	1.1 (0.5)	0.9 (0.3)^a^	5.4 (1.8)^ab^	1.2 (0.3)	1.1 (0.5)^a^	4.9 (1.7)^ab^

*Values are mean (SD) for n = 9. ^a^Significant difference to corresponding Baseline time-point. ^b^Significant difference to corresponding Pre-Ex time-point.*

### Correlation Analyses

In absolute terms, the [cotinine] correlated with performance time (*r* = 0.63, *p* = 0.05) and body mass (*r* = −0.36, *p* = 0.05) for PAT, but not for GUM (*r* = 0.04, *p* = 0.92 and *r* = −0.25, *p* = 0.29, respectively).

## Discussion

The present study sought to determine whether nicotine administration, delivered acutely via gum or more sustained via transdermal patch, proved ergogenic during a 1 h self-paced cycling TT in trained males. We used a protocol and participants that have demonstrated high validity and reliability, thereby maximizing sensitivity, and therefore these results should be applicable not only to competitive cycling but wider endurance sports/athletes. The important results are that (1) nicotine administration, regardless of delivery method, did not exert any effect (beneficial or detrimental) on exercise performance, (2) systemic delivery of nicotine was greater when using a transdermal patch than gum, and (3) nicotine administration did not affect any of the perceptual or physiological measures observed.

### Individual but Not Group Performance Is Affected by Nicotine

Several previous studies have observed a performance benefit of 7–17% when nicotine or smokeless tobacco is administered (Mündel and Jones, 2006; Johnston et al., 2018; Zandonai et al., 2018), although others have found no effect (Baldini et al., 1992; Pysny et al., 2015; Fogt et al., 2016; Zandonai et al., 2016; Mündel et al., 2017). Although all of these studies have used cycling protocols (time-to-exhaustion, 30 s Wingate, and incremental maximal tests), these are known to have poor reliability and/or validity and none have used trained cyclists. In the present study the typical error of measurement (CV) between trials was ∼3%, whilst the changes in performance attributable to either nicotine intervention was ≤1%. At an individual level, nicotine improved performance times nine times (GUM: −5.6 ± 0.8%, PAT: −4.8 ± 3.3%) compared to a detriment eleven times (GUM: +2.8 ± 1.3%, PAT: +2.8 ± 2.1%) when compared with PLA. Furthermore, nineteen of the twenty intervention trials (95%) resulted in parallel treatment outcomes i.e., both nicotine treatments collectively increased or decreased performance in the same individuals. Thus, our results indicate that in well-trained cyclists the effect of nicotine on performance is dichotomous with the effect direction dependent on the individual.

### Route of Nicotine Administration Affects Systemic Delivery

We observed that the magnitude of systemic nicotine delivery, as measured by nicotine’s major (70–80%) metabolite cotinine, was a function of route of administration (Figure 1). Whilst this is consistent with the known absorption pharmacokinetics and bioavailability of buccal versus the more sustained transdermal administration (Mündel, 2017), it is surprising that concentrations were so low with GUM (mean < 5 ng ⋅ ml^−1^). We (Mündel et al., 2017) and others (Johnston et al., 2018) have reported cotinine concentrations of 10–45 ng ⋅ ml^−1^ following administration via chewing 2 mg gum for 20 min or dispersible 5 mg sublingual strips, respectively. In the present study, of the 90 serum cotinine sample results returned, 16 (18%) were values below the limit of detection i.e., >0<7.8 ng ⋅ ml^−1^, the vast majority occurring during GUM. Consequently, it appears as though buccal absorption was not maximized and thus nicotine-rich saliva was swallowed, with subsequent first-pass metabolism. Therefore, we cannot exclude the possibility that the pharmacologic effects varied between PAT and GUM. Another explanation could be an insufficient time for the conversion of nicotine to cotinine, however this appears less likely due to the known rates of absorption and metabolism (see Benowitz et al., 2009).

Many over-the-counter products (especially pharmaceutical), require consideration whether a fixed or individualized (e.g., to body mass) dose should be administered. Results from the present study support an individualized approach if a systemic concentration determines the resultant pharmacologic effect, as lower [cotinine] was correlated with a higher body mass. Importantly, however, absolute [cotinine] correlated with absolute performance time, indicating that high(er) systemic concentrations of nicotine resulted in an *impaired* performance. At low(er) doses nicotine proves nootropic/ergogenic whilst at high(er) doses it does not (Perkins et al., 1994; Poltavski et al., 2012; Mündel et al., 2017), this dose-response relationship due to nicotine’s stimulant (low-dose), and depressant/relaxant (high doses) effects (Ashton and Stepney, 1982; Lester et al., 1988).

### Nicotine Does Not Influence Perceptual and Physiological Responses

Nicotine exerts psychostimulatory effects via increased mesolimbic dopamine, and a sympathoadrenal effect through release of the catecholamines (Mündel, 2017). However, no effects of either nicotine treatment were observed on the physiological and perceptual variables measured in the current study (Figure 3 and Table 1). This may be partly due to the self-paced nature of the exercise protocol, such that these measures reflect the relative effort and intensity of exercise i.e., power output. It has been argued previously (Mündel and Jones, 2006) that when sympathetic output is high during prolonged or high-intensity exercise, the peripheral effects of nicotine might be attenuated and the current results support this. Nevertheless, it can be seen (Figure 3 and Table 1) that the trained participants in the current study were likely close to their maximum capacity; heart rates were maintained high, by the end of exercise perception of effort was close to “Extremely Hard,” considerable hyperthermia was evident despite the temperate environment, and a reduction in bicarbonate due to a lactic acidosis had occurred.

### Considerations

Mündel (2017) proposed that in order to better interpret future results on nicotine and smokeless tobacco administration during exercise, a rigorous experimental design, for example a double-blind, placebo-control protocol with manipulation check are necessary. This is only the second study to address these shortcomings (Johnston et al., 2018), whilst the current study is the first to have sufficiently considered criterion validity of the laboratory performance test or how expert performers might respond. No study has investigated how the female response to exercise differs from men when administered nicotine or smokeless tobacco. Given that women metabolize nicotine faster than men, with this further accelerated in those taking estrogen-containing oral contraception (Benowitz et al., 2006), this provides a worthwhile avenue for investigation.

Many competitive sporting events, especially endurance-related, take place in warm-to-hot environments/climates. As a systemic venoconstrictor nicotine causes cutaneous vasoconstriction, decreased skin temperature, and systemic venoconstriction (Roth et al., 1944; Eckstein and Horsley, 1960; Benowitz et al., 1982). When combined with exercise this raises a safety concern for its use during exercise/sport with heat stress where cutaneous vasodilation and sweating are the primary routes of heat loss, potentially placing athletes at greater risk of developing a heat illness. This warrants further investigation, particularly as participants in the current study reached a T_gi_ of ∼39°C in a temperate environment.

Finally, it is worth considering the anti-doping stance. The half-life of nicotine is 1–2 h, which is why cotinine is favored as a biomarker for nicotine intake, particularly as urine samples, as its metabolism is far slower than nicotine (half-life of ∼16 h) with reduced daily fluctuation (Benowitz et al., 2009; Mündel, 2017). Therefore, it would be worthwhile comparing the detection of this WADA-monitored substance during the peri-exercise period between blood (serum) and urine indices.

## Author Contributions

TM conceived the work, obtained the funding, analyzed and interpreted the data, and wrote the first draft of the manuscript. TM, SH, and SS designed the work. All authors acquired the data and contributed to manuscript revision, read, and approved the submitted version.

## Conflict of Interest Statement

The authors declare that the research was conducted in the absence of any commercial or financial relationships that could be construed as a potential conflict of interest.
